# HEALTH COACHING IMPROVES EVERYDAY COGNITION AMONG INDIVIDUALS AT RISK FOR ALZHEIMER’S DISEASE

**DOI:** 10.1093/geroni/igad104.0410

**Published:** 2023-12-21

**Authors:** Michelle Gray, Sally Paulson, Joshua Gills, Megan Jones, Anthony Campitelli, Ray Urbina, Kelsey Bryk, Jordan Glenn

**Affiliations:** University of Arkansas, Fayetteville, Arkansas, United States; St. Elizabeth Healthcare, Edgewood, Kentucky, United States; Rutgers University, Newark, New Jersey, United States; University of Arkansas, Fayetteville, Arkansas, United States; University of Arkansas, Fayetteville, Arkansas, United States; University of Arkasnas, Fayetteville, Arkansas, United States; Neurotrack, Redwood City, California, United States; Neurotrack, Redwood City, California, United States

## Abstract

Alzheimer’s disease (AD) is the 7th leading cause of death and 6th most burdensome disease among US older adults. With pharmacological treatments for AD being predominantly ill-effective, alternative, non-pharmacological prevention and treatment strategies warrant exploration. Personal health coaching provides individualized strategies designed to improve physical, social, and emotional health leading to positive behavior changes that may improve cognitive ability. The purpose of the present investigation was to examine the effects of a Health Coaching (HC) intervention on cognitive outcomes. Adults (n=182) over 45 years of age were randomly assigned to HC or control arm of a 12-month intervention. Participants (age = 61.9 + 8.4 years) had their cognition (ECog-12) tested at baseline, 16, and 52 weeks. Participants assigned to the HC intervention met with a health coach once per month to establish healthful goals and implementation strategies improving the health and well-being of the participants. The control group received bi-weekly emails including similar information presented during the health coaching sessions. Results revealed a main effect for time for improved ECog-12 scores (p=.04) with a 3.2% improvement in ECog-12 after 52 weeks. Upon further analysis, HC had a significant improvement in cognitive performance after both 16 and 52 weeks, while the control group remained unchanged. Improvement in self-reported cognition was 4.0% (p=.03) and 5.5% (p=.01) in the HC group after 16 and 52 weeks, respectively. These results suggest HC is an effective non-pharmacological prevention strategy for cognitive decline among a group of adults at-risk for developing AD.

